# Unraveling the Core of Endometriosis: The Impact of Endocrine Disruptors

**DOI:** 10.3390/ijms26157600

**Published:** 2025-08-06

**Authors:** Efthalia Moustakli, Anastasios Potiris, Themos Grigoriadis, Athanasios Zikopoulos, Eirini Drakaki, Ioanna Zouganeli, Charalampos Theofanakis, Angeliki Gerede, Athanasios Zachariou, Ekaterini Domali, Peter Drakakis, Sofoklis Stavros

**Affiliations:** 1 Laboratory of Medical Genetics, Faculty of Medicine, School of Health Sciences, University of Ioannina, 45110 Ioannina, Greece; thaleia.moustakli@gmail.com; 2 Third Department of Obstetrics and Gynecology, University General Hospital “ATTIKON”, Medical School, National and Kapodistrian University of Athens, 12462 Athens, Greece; thanzik92@gmail.com (A.Z.); joannazouga97@gmail.com (I.Z.); ch.theofanakis@gmail.com (C.T.); pdrakakis@med.uoa.gr (P.D.); 3 First Department of Obstetrics and Gynecology, Alexandra Hospital, Medical School, National and Kapodistrian University of Athens, 11528 Athens, Greeceeirinidrak@med.uoa.gr (E.D.); kdomali@yahoo.fr (E.D.); 4 Department of Obstetrics and Gynecology, Democritus University of Thrace, 69100 Campus, Greece; agerede@otenet.gr; 5 Department of Urology, School of Medicine, University of Ioannina, 45110 Ioannina, Greece; zahariou@otenet.gr

**Keywords:** endocrine-disrupting compounds, endometriosis, chemicals, reproductive health, hormonal imbalance

## Abstract

Globally, endometriosis affects almost 10% of reproductive-aged women, leading to chronic pain and discomfort. Endocrine-disrupting compounds (EDCs) seem to play a pivotal role as a causal factor. The current manuscript aims to explain potential molecular pathways, synthesize current evidence regarding EDCs as causative agents of endometriosis, and highlight implications in the general population and clinical work. A thorough review of experimental, epidemiologic, and mechanistic research studies was conducted to explain the association between EDCs and endometriosis. Among the primary EDCs under investigation are polychlorinated biphenyls, dioxins, phthalates, and bisphenol A (BPA). Despite methodological heterogeneity and some discrepancies, epidemiologic evidence supports a positive association between some increased levels of BPA, phthalates, and dioxins in urine or in blood, and endometriosis. Experiments support some effect of EDCs on endometrial cells and causing endometriosis. EDCs function as xenoestrogens, alter immune function, induce oxidative stress, and disrupt progesterone signaling. Epigenetic reprogramming may play a role in mediating EDC-induced endometriosis. Endocrine, immunological, and epigenetic pathways link EDCs and endometriosis. Prevention techniques require deeper comprehension of those factors. Causal linkages and possible treatment targets should be based on longitudinal studies and multi-omics techniques. Restriction of EDCs could be beneficial for endometriosis prevalence limitation.

## 1. Introduction

The existence of endometrial-like tissue outside the uterus, mainly on pelvic organs such as the ovaries, fallopian tubes, and peritoneum, is a hallmark of endometriosis, a chronic, estrogen-dependent gynecological condition [1]. An estimated 190 million women worldwide suffer from the condition, which affects 10% of women of reproductive age [2]. Infertility, dysmenorrhea, dyspareunia, and incapacitating pelvic pain are clinical manifestations of endometriosis that severely reduce quality of life and place a heavy economic burden on healthcare systems around the world [3]. Although endometriosis is quite common and has a significant impact, little is known about its etiology and pathophysiology, and the only available treatments are frequently invasive surgery or symptom management [4].

Endometriosis is multicausal and multifactorial in its causation. Sampson’s theory of retrograde menstruation has long been one of the leading explanations, due to its postulation that endometriotic cells reflux through the fallopian tubes into the peritoneal cavity [5]. Up to 90% of menstruating females will have retrograde menstruation, but only a small percentage will go on to develop endometriosis. This suggests other disease susceptibility factors aside from this potential explanation, such as genetic predispositions, immunologic malfunction, hormone imbalance, as well as environmental insult [5,6]. In addition to a pro-inflammatory peritoneal milieu creating lesion formation and establishment, endometriotic implants have estrogen dominance with progesterone resistance [7].

Exposure to the environment has been given increased attention in the past few decades as a possible causative agent of increased prevalence of endometriosis. Among them, endocrine-disruptor chemicals have emerged as a focus of maximum concern [8]. By virtue of mimicking or blocking of natural hormones, EDCs are external chemicals with the potential to disrupt normal function of the endocrine system through modulation of hormone synthesis, metabolism, as well as receptor signaling [9]. These substances abound in modern life, being encountered in plastics, insecticides, cosmetic products, industrial effluents, as well as in food packaging materials. Polychlorinated biphenyls (PCBs), phthalates, dioxins, and BPA are some examples. In spite of low levels of exposures, these chemicals have the potential of bioaccumulating in adipose tissue, lingering in the environment, and causing long-term biological effects [10,11,12].

There is mounting evidence linking reproductive diseases such as endometriosis, polycystic ovarian syndrome, and infertility to exposure to endocrine-disrupting substances. Epidemiological studies have connected elevated blood or urine levels of BPA, phthalate metabolites, and dioxins to a higher incidence of endometriosis [13]. Animal studies confirm these results, showing that exposure to EDCs during pregnancy or adulthood can change the development of the reproductive tract and cause endometriosis-like lesions [13,14]. In order to promote estrogen-dependent processes that are essential to the pathophysiology of endometriosis, such as cellular proliferation, angiogenesis, and immune system dysregulation, EDCs mechanistically behave as xenoestrogens by interacting with estrogen receptors [15,16]. Emerging evidence highlights epigenetic reprogramming, whereby endocrine-disrupting chemicals alter histone modifications and DNA methylation patterns, leading to persistent changes in gene expression that facilitate lesion development [17].

Notwithstanding recent progress, key uncertainties persist concerning the causal association between endocrine-disrupting chemicals and endometriosis, the comparative significance of specific compounds versus combined exposures, and the delineation of sensitive exposure periods, including pregnancy and puberty [18]. Furthermore, methodological heterogeneity across studies has led to contradictory results, which highlights the necessity for thorough, long-term research. This includes variations in study design, exposure assessment, and population demography [19].

Given the chronic, life-altering nature of endometriosis and the widespread presence of EDCs worldwide, it is critical to comprehend how environmental exposures interact with disease pathways. Identifying at-risk groups and developing preventive measures are important but so is directing the creation of innovative treatments that target pathways mediated by the environment [20,21].

This manuscript aims to elucidate how EDCs contribute to the pathophysiology and etiology of endometriosis and to highlight the clinical and public health implications. We emphasize possible routes connecting EDC exposure to disease development; present a thorough synthesis of the most recent data from epidemiological, experimental, and mechanistic investigations; and talk about the consequences for clinical practice and public health policy. By clarifying the environmental aspect of endometriosis, we hope to promote preventive measures in reproductive health and advance a more comprehensive understanding of this mysterious illness.

## 2. Endometriosis Pathogenesis

Endometriosis is fundamentally an estrogen-dependent inflammatory disorder characterized by the formation of endometrial-like tissue outside the uterus and its ectopic implantation. Deciphering the intricate interactions between hormonal, immunological, genetic, and epigenetic elements that allow ectopic endometrial cells to survive, invade, and proliferate is essential to understanding their pathophysiology [22,23].

### 2.1. Estrogen Dependence and Hormonal Imbalance

Endometriosis is characterized by a marked dependence on estrogen, particularly estradiol, which drives the growth and persistence of ectopic lesions [24]. Endometriotic tissues exhibit elevated aromatase expression, leading to the formation of a self-perpetuating hyperestrogenic microenvironment through increased conversion of androgens to estrogens. This results in local estrogen production. The local estrogen surplus promotes cellular proliferation, angiogenesis, and apoptosis resistance, facilitating the formation and progression of ectopic lesions [25].

Concurrently, endometriotic lesions exhibit progesterone resistance characterized by diminished progesterone signaling and altered expression of progesterone receptors (PRs) [26]. Progesterone typically promotes endometrial differentiation and reduces inflammation, thereby counteracting the proliferative effects of estrogen. Prolonged estrogen-driven development and inflammation are facilitated by the decreased progesterone responsiveness of endometriotic tissue, which upsets this equilibrium [27].

### 2.2. Immune Dysregulation and Inflammation

One of the main contributing factors to the pathophysiology of endometriosis is immune system malfunction. Women with endometriosis often have changes in peritoneal immune cell populations, such as elevated levels of activated macrophages, dendritic cells, and natural killer (NK) cells. Nonetheless, a disruption in immune surveillance is often indicated by the inadequate removal of ectopic endometrial cells by these immune cells [28,29].

Tumor necrosis factor alpha (TNF-α), interleukin-6 (IL-6), and monocyte chemoattractant protein 1 (MCP-1) have been found among the cytokines and chemokines expressed in peritoneal cavity macrophages upon activation [30]. These molecules create a chronic inflammation environment, enhance angiogenesis, and maintain lesion persistence. Reactive oxygen species (ROS), generated under this inflammation condition, contribute to tissue damage as well as oxidative stress, consequently exacerbating lesion development as well as the resulting pain [31].

### 2.3. Genetic and Epigenetic Contributions

Although endometriosis is not strictly hereditary, twin studies and familial aggregation point to a genetic predisposition, with heritability estimates for the disorder ranging from 40 to 50 percent. Several susceptibility loci, including *WNT4*, *VEZT*, and *GREB1*, have been connected to genes implicated in hormone metabolism, inflammation, and cell adhesion by genome-wide association studies (GWASs) [32,33]. However, gene–environment interactions are important drivers of illness occurrence, and these genetic variants do not entirely explain it [34].

Endometriosis is increasingly understood to be influenced by epigenetic modifications, which are heritable shifts in gene expression that do not involve changes to the DNA sequence [35]. In eutopic and ectopic endometrial tissues, abnormal DNA methylation patterns, histone modifications, and dysregulated microRNAs have been found. These changes impact genes linked to extracellular matrix remodeling, immunological responses, and steroid hormone signaling [36]. Endocrine disruptors and other environmental exposures may have an impact on these epigenetic modifications, which could mediate their effects on the onset of disease [37].

### 2.4. Angiogenesis and Tissue Invasion

Neovascularization is necessary for the development of endometriotic lesions to provide oxygen and nutrients. Vascular endothelial growth factor (VEGF), which promotes the formation of new blood vessels and aids in lesion survival, is one of the angiogenic factors that are produced in considerable amounts by endometriotic tissues [38,39]. Furthermore, ectopic endometrial cells express more matrix metalloproteinases (MMPs), which degrade extracellular matrix components to facilitate tissue invasion and adhesion creation [40].

**Figure 1:**
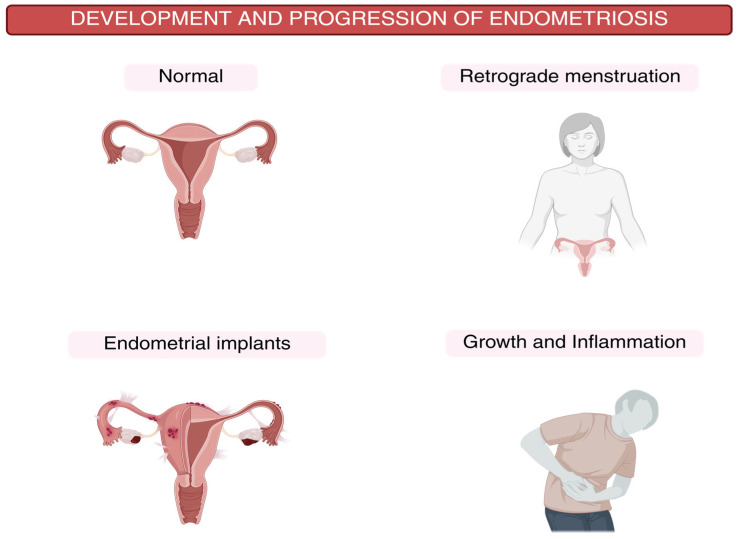
Schematic representation of the development and progression of endometriosis.

The chronicity and advancement of endometriosis are facilitated by the combined effects of increased angiogenesis and invasive capacity, which allow ectopic lesions to implant and persist in ectopic locales [41]. Table 1 summarizes the key pathogenic mechanisms of endometriosis and Figure 1 illustrates the development and progression of endometriosis.

## 3. Endocrine Disruptors: Definition and Mechanisms

**Figure 2:**
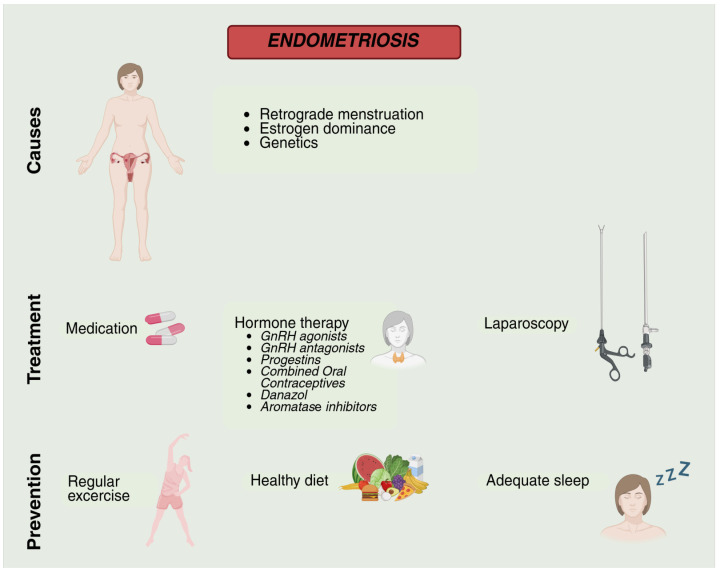
Overview of endometriosis, including potential causes, treatment options, and preventive measures.

EDCs are a seemingly unrelated set of exogenous chemicals that cause interference in normal endocrine function, for instance, hormone synthesis, metabolism, and signaling [42]. Reproductive function, growth, development, and homeostasis require the fine-tuned balance in hormones offered by the endocrine system. Endometriosis and reproductive disorders are significant adverse health outcomes of the disruption in endocrine homeostasis caused by exogenous or endogenous chemicals [43]. Figure 2 provides an overview of potential causes and preventive measures of endometriosis.

### 3.1. Definition and Sources of Endocrine Disruptors

Exogeneous chemicals or mixtures, also referred to as EDCs, are known to interfere with normal endocrine function and can even have adverse effects on people’s or populations’ health [44]. The United Nations Environment Programme (UNEP) and the World Health Organization (WHO) uphold such an idea, which emphasizes means through which the EDCs would modify hormonal signaling pathways for crucial physiological processes like development, reproduction, and metabolism. The below are means through which the EDCs would modify hormone synthesis and metabolism, antagonize hormone receptors, mimic endogenous hormones, and modify receptor expression and downstream signaling processes [45].

As their use in consumer products, industries, and agriculture is quite high, their presence is widespread in the environment. Industrial chemicals like PCBs, dioxins, and polybrominated diphenyl ethers, often used as flame retardants and in electrical appliances, are a leading cause of environmental pollution [46]. Plasticizers like BPA, contained within epoxy resins and polycarbonate resins, and phthalates, which are in use for the convenience of plastic flexibility, are greatly used in consumer products as well as in packaging for food [10]. Pesticides such as atrazine and dichlorodiphenyltrichloroethane (DDT), as well as body-care ingredients like parabens, triclosan, and ultraviolet filters, are also recognized EDCs with widespread exposure potential [47].

Dietary consumption is a substantial pathway of EDC exposure in addition to environmental and consumer product exposures. Naturally occurring phytoestrogens that have estrogenic properties and can affect hormonal balance include genistein and coumestrol, which are frequently found in soy products, flaxseed, and legumes. Depending on the situation and the individual, these substances may play both protective and disruptive roles. Additionally, tainted food, agricultural waste, and leaching from food packaging materials can all introduce synthetic EDCs into the diet. Dietary exposure is persistent; thus, it should not be disregarded for its contribution to the cumulative EDC burden and possible influence on hormone-sensitive diseases such as endometriosis.

Since these compounds are persistent, bioaccumulative, and widely used, they are constantly present in human bodies through ingestion, inhalation, skin contact, and even transplacental transfer during pregnancy [48,49,50].

### 3.2. Mechanisms of Endocrine Disruption

Endocrine disruptors interfere with the normal activity of hormones through a variety of molecular and cellular mechanisms. Direct binding to hormone receptors is one of the main ways that interference occurs [51]. Numerous EDCs have agonistic or antagonistic effects on nuclear receptors, including thyroid hormone receptors, androgen receptors (AR), and estrogen receptors (ERα and ERβ). The balance of hormone signaling can be upset, for instance, when BPA and a number of phthalates bind to estrogen receptors and either impede receptor function or excessively activate estrogen-responsive genes [52,53].

In addition to affecting receptor binding, endocrine-disrupting chemicals can alter hormone synthesis and metabolism by modulating enzymes critical to steroid hormone biosynthesis and degradation [54]. These include modulation of hormone-clearing cytochrome P450 enzymes, as well as 5α-reductase and aromatase, the enzyme that transforms androgens into estrogens. Such alterations can lead to local and systemic changes in hormone levels, thereby potentially worsening hormone-regulated disorders [55]. Moreover, EDCs can cause epigenetic modifications like DNA methylation, histone acetylation, and changes in miRNA production. The impact of exposure to endocrine-disrupting chemicals may be exacerbated by such epigenetic changes, which may have long-lasting and heritable impacts on gene expression across generations [56,57].

Additionally, by interfering with the blood’s hormone transport proteins, endocrine disruptors affect the availability and delivery of hormones to their target regions [58,59]. Systemic hormonal changes affecting various organs and bodily systems can result from endocrine-disrupting chemicals that interfere with hormone feedback loops, particularly in the hypothalamic–pituitary–gonadal axis. The complex and dynamic processes by which endocrine-disrupting chemicals impact endocrine function further suggest their likely role in hormone-dependent disorders such as endometriosis [60,61].

## 4. Evidence Linking Endocrine Disruptors to Endometriosis

Mounting clinical, experimental, and epidemiologic evidence supports a significant link between exposure to endocrine-disrupting chemicals and the development and continuation of endometriosis [62]. The evidence introduces the prospective role for endocrine-disrupting chemicals in the pathophysiology of the multifactorial disease through disruption of hormone signaling, immune function, and epigenetic regulation. Individuals suffering from endometriosis are demonstrated to harbor higher concentrations of persistent organic pollutants (POPs), namely dioxins, PCBs, and some selected pesticides, in comparison to control groups in various epidemiologic analyses [63]. The link verifies the hypothesis that such lipophilic chemicals become concentrated within adipose tissue as well as in peritoneal fluid and exert local activity on an estrogenic as well as immunomodulatory basis; for example, higher concentrations of serum chemicals exhibiting dioxin-like activity are associated with risk and severity of endometriosis [64].

Similarly, studies have reported higher concentrations of BPA and phthalates in the urine or serum of women with endometriosis, implicating these more transient yet prevalent endocrine-disrupting chemicals in the disease’s development [65].

This association is further corroborated by experimental animal models demonstrating that exposure to endocrine-disrupting chemicals during pregnancy or early life may predispose offspring to endometriosis-like lesions in adulthood [21]. Rodent studies have demonstrated that low-level exposure to BPA or dioxins during critical periods of reproductive system development alters immune responses, hormone sensitivity, and gene expression, thereby promoting the formation of ectopic endometrial tissue [66]. These results are consistent with the theory of developmental reprogramming, which holds that environmental shocks experienced early in life have long-term effects on reproductive health.

At the molecular level, in vitro research shows that EDCs can increase the expression of VEGF, which promotes angiogenesis, and they can also stimulate the migration, invasion, and proliferation of endometrial stromal and epithelial cells [38]. Additionally, it has been demonstrated that EDCs alter the production of MMPs, which are important enzymes that promote lesion implantation and tissue remodeling. Additionally, exposure to EDCs affects the behavior of immune cells, changing NK cell activity and boosting the secretion of pro-inflammatory cytokines, which adds to the chronic inflammation associated with endometriosis [67,68].

EDC-induced epigenetic modifications indicate another mechanistic connection. Studies have shown that endometrial cells exposed to EDC have aberrant histone alterations and DNA methylation patterns in genes linked to immune regulation and hormone signaling. These epigenetic changes may be responsible for the progesterone resistance and estrogen dominance observed in endometriotic lesions [69,70].

Another crucial mechanism linking EDCs to endometriosis involves the xenobiotic biotransformation system, which is responsible for metabolizing and detoxifying foreign compounds [71]. EDCs are modified by oxidation by Phase I enzymes (like cytochrome P450s, especially CYP1A1 and CYP1B1) and then conjugated by Phase II enzymes (such as glutathione S-transferases, N-acetyltransferases, and UGTs) to improve solubility and simplify excretion. These detoxifying enzymes’ genetic variations may have a major impact on a person’s ability to eliminate EDCs [72]. For instance, higher endometriosis risk and increased EDC burden have been linked to reduced-function alleles in *GSTT1* and *GSTM1*, most likely as a result of decreased clearance of toxic metabolites [73]. Furthermore, the response to dioxin-like substances may be further hampered by dysregulation of the aryl hydrocarbon receptor system, which controls the expression of numerous detoxification genes. These results underline the significance of gene–environment interactions in disease development by indicating that interindividual genetic diversity in xenobiotic metabolism may alter susceptibility to endometriosis after EDC exposure [74].

Despite mounting evidence, disparities continue to exist among studies, most likely as a result of the complexity of endometriosis, research population heterogeneity, and variations in exposure assessment techniques. Reducing environmental exposures as part of preventive measures is crucial, and the combination of results from cellular, animal, and human investigations provide strong evidence for the involvement of endocrine-disrupting chemicals in the etiology of endometriosis [75].

## 5. Potential Mechanisms of EDC-Induced Endometriosis

EDCs contribute to the development and progression of endometriosis by disrupting normal hormonal, immunological, and cellular processes that are necessary for preserving uterine and peritoneal homeostasis. Understanding these mechanisms is crucial to comprehending how environmental exposures lead to the pathophysiology of disease [13,70].

Estrogenic action is among the major mechanisms. The majority of EDCs act by binding to ERα and ERβ estrogen receptors of endometrial and peritoneal cells in order to resemble estrogen’s structure as well as activity [76]. Uncontrolled endometrial growth outside of its normal location defines endometriosis, and this is encouraged by this aberrant stimulation of estrogen action [77]. Moreover, in endometriotic lesions, there is potential for EDCs to upregulate expression of aromatase, this enzyme being responsible for androgen conversion to estrogens. This would locally promote estrogen production and create an estrogen-rich milieu that would encourage lesion propagation as well as persistence [78,79].

Another crucial mechanism is progesterone resistance. Progesterone’s normal anti-proliferative effects on the endometrium are disrupted by this condition, which is common in women with endometriosis. Exposure to EDC has been shown to alter progesterone receptor expression and signaling pathways, thereby exacerbating unopposed estrogenic effects and contributing to lesion persistence [80,81].

Immune dysregulation induced by EDCs represents a key contributor to the pathogenesis of endometriosis. The immune system typically facilitates the clearance of retrograde menstrual debris and prevents the ectopic implantation of endometrial cells [82]. However, by changing the activity of T lymphocytes, NK cells, and macrophages, EDCs can disrupt immune cell function, resulting in a pro-inflammatory environment and decreased clearance of ectopic tissue. This persistent inflammation exacerbates endometriosis-associated pain and fibrosis while sustaining lesion progression [83,84].

EDC also contribute to pathogenesis through the induction of OS. Enhanced ROS generation facilitates the development and persistence of endometriotic lesions by damaging cellular components, activating inflammatory signaling pathways, and promoting angiogenesis. Furthermore, OS can result in epigenetic changes and DNA damage, which might prolong aberrant gene expression [85].

Epigenetic changes are a crucial and newly discovered mechanism via which endocrine-disrupting drugs affect endometriosis. EDCs have been demonstrated to induce changes in DNA methylation, histone modifications, and microRNA expression in endometrial cells [70,86]. By disrupting the regulation of genes involved in immune response, hormone responsiveness, and cell proliferation, these epigenetic changes may result in a cellular phenotype characteristic of endometriotic tissue. The chronic nature of the disease and its familial occurrence may be attributed to the possibility that these changes are both permanent and heritable [87,88].

Furthermore, endometriosis susceptibility is modulated by genetic factors that control sex hormone levels, such as polymorphisms in genes encoding steroidogenic enzymes (CYP19A1 and HSD17B1), estrogen receptors (ESR1 and ESR2), and hormone-binding proteins [89]. Variants in these genes have been linked to changes in estrogen metabolism, receptor function, and local hormone bioavailability by recent GWASs and candidate gene analyses [90].

In addition to these, recent large-scale GWASs and associative studies have identified several other genetic loci implicated in endometriosis risk, including follicle-stimulating hormone beta subunit (*FSHB*), luteinizing hormone/choriogonadotropin receptor (*LHCGR*), *SYNE1*, and genes related to age at menarche [32,91,92,93]. These genes are involved in hypothalamic–pituitary–ovarian axis regulation and the timing of reproductive maturation, both of which are critical for endometrial homeostasis and disease development.

Epigenetic processes, such as modifications in histone proteins, increased chromatin accessibility, and alterations in transcription factor binding at hormone-responsive loci, are frequently used to achieve these genetic consequences [94]. Polymorphic alleles implicated in estrogen and progesterone signaling or aberrant expression of gene regulatory networks might result from exposure to EDCs, which can interfere with these very processes. For instance, it has been demonstrated that BPA and dioxins increase histone acetylation or change DNA methylation at the promoter regions of genes linked to hormones [70].

Emerging evidence suggests that EDCs may also interact with regulatory regions of *FSHB*, *LHCGR*, *SYNE1*, and other reproductive-hormone-associated genes, influencing their transcriptional activity and amplifying hormonal dysregulation [91,93]. This could augment or decrease the phenotypic impact of previously mild or dormant genetic variants, which would lead to progesterone resistance, estrogen dominance, and heightened susceptibility to endometriosis. These findings highlight a critical gene–epigenetic–environment interaction in the pathogenesis of the disease [35,95].

These pathways demonstrate that exposure to endocrine disruptors can both initiate and perpetuate the complex pathological processes that underlie endometriosis. These pathways are presented in Table 2 and need to be clarified more in order to create focused preventative and treatment plans [21].

## 6. Clinical Implications and Future Directions

To treat and prevent endometriosis, there is growing evidence that endocrine disruptors are linked to the disease. The discovery that endocrine-disrupting chemicals contribute to the pathophysiology of endometriosis underscores the importance of environmental health considerations in reproductive medicine, as well as the need for integrated therapeutic approaches for patients [18].

Decreases in exposures to proven endocrine disruptors would lower endometriosis prevalence aimed at sensitive developmental points like early life as well as pregnancy [8]. This would enlist public health actions in enhancement of awareness as well as in advocacy for regulation of endocrine disruptive chemicals in industrial effluents, food packaging materials, as well as consumer products. Health practitioners must be informed of sources of endocrine disruptive chemical exposures and recommend measures of decreasing such exposures to those of reproductive age, in particular [96].

Recognizing endocrine-disrupting chemical exposure as a contributing factor in diagnosis and treatment may facilitate the development of novel biomarkers to improve early detection and predict disease severity [97]. Personalized risk assessment could be enhanced through biomonitoring of specific endocrine-disrupting chemicals or related molecular signatures in biological fluids. Furthermore, determining the molecular pathways via which EDCs affect endometriosis may help find novel targets for treatment [98]. Adjunctive therapeutic approaches could include medications that counteract hormone receptor dysregulation brought on by EDCs or modify epigenetic alterations [86].

To fill in the current information gaps, future research is crucial. This includes studies that examine the combined effects of various EDCs and other environmental factors, standardize exposure measurement, and establish causality through longitudinal studies [99]. Technological developments in omics, epigenetic profiling, and high-throughput screening will improve our knowledge of the molecular and systemic mechanisms by which EDCs contribute to endometriosis [100].

Translating new information into successful therapies ultimately requires an interdisciplinary strategy involving researchers, doctors, public health authorities, and legislators. Endometriosis may be lessened, and the quality of life for afflicted women around the world may be enhanced by including environmental health into reproductive care [101].

## 7. Future Directions

Despite significant advances in understanding the relationship between endocrine-disrupting chemicals and endometriosis, many critical questions remain unresolved. We believe that continued and targeted research is essential to unravel the complex interactions among genetic predisposition, environmental exposures, and hormonal regulation that contribute to the onset and progression of the disease.

Improving and standardizing exposure assessment techniques is one of the top priorities. Current epidemiological studies often rely on single-point measurements of EDCs in biological samples, which may not accurately reflect cumulative or long-term exposures. We recommend that future research employ longitudinal designs with repeated sampling and include more detailed exposure profiles that consider combined and mixed EDC exposures. This approach could facilitate the verification of causation and dose–response relationships.

Additionally, using omics and molecular biology approaches to elucidate how EDCs impact endometriosis holds great promise. Researchers may identify biomarkers of exposure, susceptibility, and disease development by combining transcriptomics, metabolomics, epigenomics, and genomics. To test theories and evaluate potential therapies, we support the development of improved animal models that more closely resemble human disease phenotypes and real exposure conditions.

Examining the effects of EDC exposure on generations is another important area. Recent data suggests that these chemicals may cause heritable epigenetic changes, potentially increasing disease risk in offspring. To improve early intervention and prevention, we recommend that future research focus on understanding the mechanisms behind these transgenerational effects.

To address the molecular effects of EDC exposure, we advise investigating therapeutic approaches, including reversing epigenetic changes or modifying hormone receptor signaling pathways. Furthermore, as supplementary methods to current treatment approaches, we think that dietary and lifestyle treatments targeted at lowering the body burden of EDCs or lessening their effects should be studied.

Finally, we stress the importance of interdisciplinary cooperation between environmental scientists, public health specialists, toxicologists, and physicians. To lessen exposure, especially among susceptible groups like adolescents and pregnant women, we support public education campaigns. It should be a shared priority to translate research findings into practical preventive measures and regulatory legislation.

Pursuing these research directions may enable the medical and scientific communities to gain a deeper understanding of the role of endocrine disruptors in endometriosis and ultimately contribute to improved outcomes for affected individuals.

## 8. Conclusions

Endometriosis is a complicated, multifaceted illness with significant causal agents that include hormonal, immunologic, and environmental variables. The importance of EDCs in the pathophysiology is becoming more and more clear [102]. Progesterone resistance, immunologic dysregulation, oxidative stress, estrogenic mimicry, and epigenetic modifications are some of the ways that EDCs disturb the delicate hormonal balance necessary for healthy endometrial function. This facilitates the development and establishment of lesions [70].

Beyond understanding disease causation, understanding these mechanistic associations further emphasizes relevance in incorporating environmental exposure as a cornerstone in effective endometriosis prevention and treatment measures. Decrease in human exposure to negative EDCs and improvement in measure of exposure, in addition to provision of research into selective therapies, can alleviate endometriosis disease burden. Improving reproductive health worldwide and protecting vulnerable populations will entail incorporating environmental health considerations in bedside care and policy as knowledge advances. Identifying endocrine disruptors’ essential effects thus presents a possible means of decreasing the prevalence and severity of endometriosis as well as improving the quality of life of affected individuals.

## Figures and Tables

**Table 1:** Key pathogenic mechanisms in endometriosis. This table summarizes the principal biological processes involved in the development and progression of endometriosis.

Pathogenic Mechanisms	Description	Key Factors Involved	Role in Disease
Estrogen Dependence	Local overproduction of estrogen promotes lesion growth and survival	Aromatase, estradiol (E2)	Drives proliferation, angiogenesis, and inhibits apoptosis
Progesterone Resistance	Impaired progesterone receptor expression leads to reduced anti-inflammatory and differentiation effects	Progesterone receptor (PR)	Allows unchecked estrogenic effects and inflammation
Immune Dysregulation	Altered immune cell function impairs clearance of ectopic cells and promotes inflammation	Activated macrophages, cytokines (TNF-α, IL-6), NK cells	Creates pro-inflammatory environment, sustains lesion growth
Oxidative Stress (OS)	Reactive oxygen species induce tissue damage and promote inflammation	ROS, OS markers	Exacerbates inflammation and lesion progression
Genetic Susceptibility	Inherited variants increase vulnerability to endometriosis	GWAS-identified genes (*WNT4*, *VEZT*, *GREB1*)	Modulates hormonal, immune, and adhesion pathways
Epigenetic Alterations	DNA methylation and histone modifications alter gene expression affecting key pathogenic pathways	DNA methylation, microRNAs, histone modification enzymes	Sustains disease by modulating hormone signaling and immunity
Angiogenesis	Formation of new blood vessels to supply ectopic lesions	VEGF, angiopoietins	Supports lesion survival and expansion
Tissue Invasion	Degradation of extracellular matrix enables implantation and adhesion	MMPs	Facilitates lesion implantation and spread

**Table 2:** Potential mechanisms by which EDCs contribute to the pathogenesis of endometriosis. This table outlines key biological processes affected by EDC exposure, including estrogenic activity, progesterone resistance, immune dysregulation, oxidative stress, and epigenetic alterations.

Mechanism	Description	Examples of EDC Effects
Estrogenic Activity	EDCs mimic or enhance estrogen signaling by binding estrogen receptors and increasing local estrogen production.	Activation of ERα/ERβ by BPA and phthalates. Upregulation of aromatase in lesions increasing local estrogen.
Progesterone Resistance	EDCs disrupt PR expression/signaling, reducing progesterone’s regulatory effects on the endometrium.	Impaired PR expression leading to unopposed estrogen action, promoting lesion growth and persistence.
Immune Dysregulation	EDCs alter immune cell function, impairing clearance of ectopic tissue and promoting inflammation.	Reduced NK cell cytotoxicity, altered macrophage activation, increased pro-inflammatory cytokines.
OS	EDC-induced ROS cause cellular damage, inflammation, and promote angiogenesis.	DNA damage and activation of inflammatory pathways contributing to lesion establishment and fibrosis.
Epigenetic Alterations	EDCs cause changes in DNA methylation, histone modifications, and microRNA expression, affecting gene regulation.	Altered gene expression related to hormone response and immune function. Stable and heritable modifications.

## Data Availability

No new data was created or analyzed in this study. Data sharing is not applicable to this article.
